# Feasibility study of the Digital Patient Benefit Assessment Scale (P-BAS): A Digital Tool to Assess Individual Patient Goals

**DOI:** 10.1177/23337214241230159

**Published:** 2024-02-07

**Authors:** Rozemarijn A.L. van Erp, Sophia E. de Rooij, A.N. Machteld Wymenga, A.V.C.M. Elgun Zeegers, Job van der Palen

**Affiliations:** 1 University of Twente, Enschede, The Netherlands; 2 Medisch Spectrum Twente, Enschede, The Netherlands; 3 University of Groningen, University Medical Center Groningen, University Center for Geriatric Medicine, Groningen, The Netherlands

**Keywords:** older patients, feasibility, goal-setting, shared decision making, digital

## Abstract

The study objective was to assess the feasibility of the Patient Benefit Assessment Scale (P-BAS), a digital tool designed to enable older outpatients (≥70 years) to elucidate at home their individual goals regarding their current medical issue. Several digital tools are developed to assist older people in identifying their goals, thereby facilitating the process of shared decision making. However, studies on the feasibility of these digital tools, especially in older patients, are limited. Data were collected from 36 older patients. The study comprised three stages. In stage I and II, cognitive interviews were conducted to strengthen the feasibility of the P-BAS. In stage III, 80% of the patients completed the P-BAS independently at home. The cognitive interviews provided insight into patients’ interpretation and individual understanding of the digital visual P-BAS and associated opportunities for improvement, which were subsequently implemented. One conclusion is that the digital visual P-BAS might be of added value for patients and contributes to the process of shared decision making, assuring that the goals of the patient will be into account in treatment options. Findings are useful for researchers interested in technological tools that contribute to shared decision making.

## Introduction

The worldwide population is aging, and so is the number of patients with multiple chronic diseases (van der Kluit et al., 2019). Disease-oriented care assessment focuses on disease-specific outcomes such as control of hypertension and regulation of diabetes (Tinetti et al., 2004). Although these outcomes might work well for relatively healthy patients with single diseases, they may be inappropriate for patients with multiple conditions, facing decline of severe disability, or a short life expectancy (Reuben & Tinetti, 2012; Tinetti, Naik, & Dodson, 2016). Older patients (aged ≥ 70 years) with multimorbidity may be more interested in personal goals such as alleviation of their disabling symptoms and improvement in social and physical functioning, rather than group-level outcomes such as mortality and survival (van der Kluit et al., 2019; N. P. Vermunt et al., 2018). Therefore, a shift is recommended from disease-oriented toward a more goal-oriented care approach (Reuben & Tinetti, 2012; Schulman-Green et al., 2006; Tinetti, Naik, & Dodson, 2016; van der Kluit et al., 2019; N. P. Vermunt et al., 2018). The consequences of treatment options and the growing demand for goal-oriented care and shared decision making especially in the highest age groups, increasingly question whether treatment options, as suggested by healthcare professionals, always align with the preferences, goals and needs of older adults (Reuben & Tinetti, 2012; Tinetti, Esterson et al., 2016). The more appropriate perspective would be to focus on patient’s individual health goals within or across a variety of dimensions and determine how well these goals are being met.

Goal-oriented care has several important advantages. Firstly, it motivates patients to increase focus and awareness of goals they want to achieve while receiving the treatment (Schulman-Green et al., 2006). Based on these individual goals and preferences of older patients the added value of a specific treatment choice can be assessed, whereby in some cases refraining treatment also becomes an acceptable option.

Secondly, for healthcare professionals, the opportunity arises to discuss and anticipate on these goals in order to identify the proper treatment. According to Mulley et al. (2012) an accurate medical diagnosis alone is not sufficient to identify the best treatment. At least as important is an accurate preference diagnosis, meaning a diagnosis of the priorities, preferences and goals of the patient himself. Mulley et al. (2012) shows that misdiagnosing patients’ preferences are commonplace. What patients want often differs from what doctors think they want.

Thirdly, goal-oriented care contributes to an effective, tailor-made shared decision-making process. The patient selects the health outcome with the highest priority and the clinician determines what treatment strategies are most likely to achieve that outcome.

Discussing individual goals of a patient can guide decision-making in case of multimorbidity and provides important information for handling health situations in the future (Elwyn & Vermunt, 2020; van der Kluit et al., 2018, 2019; N. Vermunt et al., 2019). Besides, shared decision-making can result in an increased achievement of positive health outcomes through well balanced clinical interventions. Examples of these health outcomes include better adherence to medication, improved disease management, and improved functioning after rehabilitation (Schulman-Green et al., 2006). The focus on desired outcomes may also contribute to lower health costs, if achieving desired outcomes requires fewer resources than traditional disease-oriented care (Berwick et al., 2008; Mold, 2017; Reuben & Tinetti, 2012).

Participation in shared decision-making and aligning treatment strategies toward more common and desirable goals, can be challenging for older patients (Bunn et al., 2018; Schulman-Green et al., 2006). Therefore, tools are developed in order to facilitate the process of shared decision-making between patients and their healthcare professionals. Some of these tools are specifically designed to help older patients identify and prioritize their goals to facilitate the shared decision-making process (Tinetti et al., 2018).

The Patient Benefit Assessment Scale (P-BAS) is a recently developed digital picture-based tool to identify the personal goals of older acutely hospitalized patients (van der Kluit et al., 2019). The P-BAS could be most valuable when used before hospital admission or specific treatment takes place. The goals of the patient can be discussed between patient and healthcare professional and are taken into account before the treatment takes place. Refraining from treatment would become in some cases also an acceptable treatment. This may add value to the shared decision-making process. A better shared decision-making process whereby goals of patients are taken into account by the healthcare professional, during the consult with the patient, can lead to the best personalized care for the patient, positive health outcomes (adherence to medication) and a possible reduction of health costs. It is however unclear if patients are able to use this digital tool without support in the attendance of a health care professional. The objective of this study is therefore to assess the feasibility of the digital P-BAS in patients receiving specific treatment. This leads to the research question: “Are patients able to independently fill in the digital visual P-BAS at their home without the assistance of a health care professional?”

## Methods

This qualitative study was conducted by inviting patients from the departments of medical oncology and orthopedics of Medical Spectrum Twente (MST), a large teaching hospital in the Netherlands, to participate in cognitive interviews (Priede & Farrall, 2011). For this research a full review of the research protocol according to the Dutch Medical Research with Human Subjects Law (WMO) was not applicable since, the participants are not subjected to procedures or are required to follow rules of behavior which are an infringement of the physical and/or psychological integrity of the subject. The research protocol was approved by the Institutional Review Board of Medisch Spectrum Twente (K21-14). A semi-structured interview scheme was created for asking clarifying, deepening and evaluating questions during the cognitive interviews (Willis, 1999).

Patients were selected by their treating physicians from the departments of medical oncology and orthopedics. The focus was on these patient groups because of the range of treatment options available for these conditions. After selection patients were approached by phone for participation. Patients were selected until data saturation was reached. The inclusion criteria for participation consisted of (1) age ≥ 70 years (de Rooij et al., 2009), (2) capable of speaking and understanding Dutch, (3) visiting the hospital for an outpatient consultation, (4) in possession of an e-mail address, and (5) an active care demand for breast cancer, hip, or knee osteoarthritis. One exclusion criteria was cognitive limitations (e.g., dementia or Alzheimer’s disease) since this could influence the awareness of their cognitive functions (Giannouli & Tsolaki, 2022). All patients were asked for written informed consent before participating in the study.

The researchers with a background in Health Sciences/Public Management and familiar with interviewing techniques and in performing qualitative research were trained by the principal investigator in the cognitive interviewing technique and to ask the structured questions. Test interviews were performed in order to improve the cognitive interviewing technique (Willis, 1999).

### Procedures

A three stage method was used in order to test the feasibility of the P-BAS. The pictures of the 22 predefined goals [insert Appendix 1] were digitalized in a questionnaire with the use of the program CastorEDC. For each goal the patient was asked to indicate whether or not the goal is applicable/important for them (e.g., “Is doing groceries a goal?”) When the patient defined a goal as important, they received three additional questions in which patients were asked to rate (1) the level of importance of that goal on a three point Likert scale (somewhat, fairly, very much), (2) what they expected to achieve through the treatment (maintain the goal or improve the goal), and (3) how they were feeling regarding that goal right now (six point Likert scale from very bad to vary good). After each stage, evaluation took place and relevant changes in the P-BAS were implemented.

### Stage 1. Cognitive Interviews

In stage I cognitive interviews in Medical Spectrum Twente (MST) were carried out in order to identify problems in filling out the digital visual P-BAS. After written informed consent was obtained, the researcher provided the patient with the digital pictured based version of the P-BAS. During the cognitive interviews, patients had to complete the P-BAS. The researcher classified the goals of the questionnaire that caused problems or ambiguities into a classification scheme (see Table 1) (Buers et al., 2014; Willis, 1999, 2004). A second researcher observed the interview and independently classified the goals of the questionnaire that caused problems or ambiguities into the classification scheme as well. After the interview, the two researchers discussed their classifications and reasoning for choosing certain categories. Eventually, one classification scheme with the same identification of problems was the result.

**Table 1.** Classification Scheme for Identifying Improvements for P-BAS.

Category	Explanation
Clarity	Problems with the intent or the meaning of a question (e.g., due to difficult or vague words/formulations)
Knowledge	Likely to not know or have trouble remembering information
Assumptions	Problems with assumptions or underlying logic
Response categories	Problems with the response categories, due to the lack of an appropriate answer to the question, or due to vague, overlapping or insufficient ordered answer options, or due to a mismatch between question and answer, or due to insufficient disease-specific goals
Sensitivity	Sensitive nature or wording/bias
Instructions	Problems with introductions, instructions, or explanations
Formatting	Problems with layout or question ordering

The methods of thinking aloud and verbal probing where used by the researcher during the interview while the participant filled out the P-BAS (Beatty & Willis, 2007; Presser et al., 2004; Willis, 2004). When the patient had completed the P-BAS, the researcher asked evaluating questions (Text Box 1 below). All interviews were recorded and useful remarks by the participants were identified and analyzed. The answers from the evaluating questions of P-BAS were transcribed verbatim. Based on those notes, the classification scheme and transcripts, further possible improvements for the P-BAS were identified and implemented.

**Text Box 1.** Evaluating Questions Stage I.

Evaluating questions stage I
• Was the instruction for the digital P-BAS clear?
○ Was there any information you were missing?
○ Was it clear for you what was expected from you with the digital P-BAS?
• What do you think of the topics in the digital P-BAS?
○ Where there any missing topics? If yes, which topics?
• How well is the digital P-BAS reflecting your own goals?
• What did you think of the length of the digital P-BAS?
• What would you prefer: filling in the digital P-BAS at home (alone or together with your loved ones) or in the hospital together with a nurse/doctor?
• Would you be able to fill in the digital P-BAS at home? If no, what do you need?
○ Would an e-mail with a link to the digital P-BAS suffice?
• Could you be helped at home if things in the digital P-BAS are unclear?
○ Who could you ask for help?

### Stage II. Completing the P-BAS in the Hospital and at Home

In stage II patients completed the P-BAS in MST, again using the cognitive interview method. After 3 to 5 days the patients received an e-mail link to fill out the P-BAS again, in order to test whether the patients were able to use a link and complete the P-BAS independently at home. After completing the P-BAS at home, patients were called for additional evaluating questions (Text Box 2). Based on the transcripts, the classification scheme, and answers on the evaluating questions, improvements for the P-BAS were implemented.

**Text Box 2.** Additional Evaluating Question Stage II and III.

Additional evaluating questions stage II and III
• How was your experience regarding receiving and opening the e-mail and thereby the digital P-BAS?
○ What changes would you recommend to make this experience easier?
• What was your experience about the instruction video?
○ Was it clear for you after seeing the instruction video what was expected?
• Did you receive any help from someone in completing the digital P-BAS?
○ If yes, who did you receive help from?
• You have now completed the questionnaire at home, did you like this or would you rather do it together with the nurse/doctor?

### Stage III. Completing the P-BAS at Home

In stage III the patients only received a link to fill out the P-BAS remotely. After filling in the P-BAS at home, patients were called for additional evaluating questions (Text Box 2).

## Results

**Figure 1.**
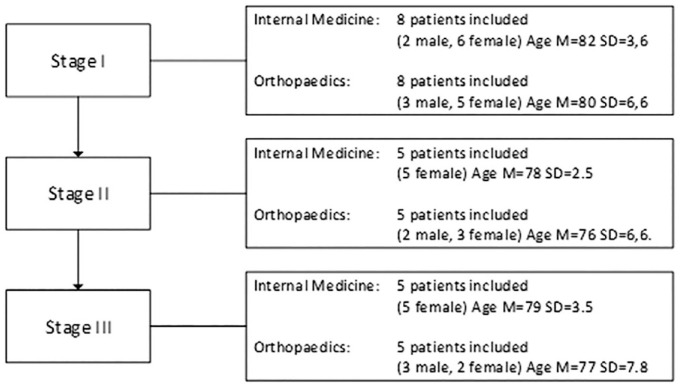
Patients demographics.

In total 36 patients were included (see Figure 1).

Analysis of the cognitive interviews of 16 patients of stage I, revealed 181 problems within the P-BAS tool [insert Appendix 2]. A large number of problems was observed in the category “clarity” of the goals, which was mainly due to the formulation of the questions. For example: “Is pain a goal?” a patient responded (*patient 5 oncology stage I)*: “this question makes no sense, of course it is not a goal to have pain, no one wants that.” Another example is the goal feeling better were a patient *(patient 4 orthopedics stage I)* responded: “Is feeling better a goal?. . . What do I need to fill in if I am feeling good at the moment? Everyone wants to feel better I think.”

Based on these findings, the formulation of seven goals was adjusted for example, “Is preventing or reducing pain a goal?” The categories “assumptions” and “response categories” also revealed a relatively large number of problems. To address these issues, the introductory text has been adapted to make it clearer that the P-BAS tool is about the patient’s goals regarding their hospital treatment. In addition, an instruction video was added to the introduction. The last improvement was the closing text. The text contained after adjustment more specific instructions about completing and closing the P-BAS tool.

In Stage II, all participants (*N* = 10) completed the digital P-BAS in MST and at home. Stage II revealed a total number of 70 problems [insert Appendix 3] within the P-BAS tool. Goals with five or more problems were selected and further discussed for possible improvements. Regarding the category “clarity” the formulation of several goals and follow-up questions was adjusted. Concerning the categories “assumptions” and “response categories” the lay-out of the questions and the introduction text were adjusted. The introduction text contained after adjustment some specific examples and instructions about filling out the digital P-BAS.

In stage III ten patients received a link of the digital P-BAS at home. Eight patients fully completed the digital P-BAS. One patient *(patient 16 orthopedics stage III)* started with the digital P-BAS but did not complete the entire questionnaire. Although several attempts were made to call the patient and ask for an explanation for not completing the questionnaire, the researchers were unable to reach the patient. Another patient *(patient 16 oncology stage III)* was not able to fill out the P-BAS at home due to technical reasons. “I am not sure if I gave you the right e-mail address. . . but I also do not know how the inbox of my e-mail works. I am not really good in all these technical things.” After filling out the digital P-BAS patients were called by telephone. Based on the evaluating questions and the previous findings of stage I and II, the order of the goals in the P-BAS was adjusted. Next to this the goal “feeling better” was removed from of the questionnaire. This goal was positively answered by all patients, and was not a distinguishing question. The data that support the findings of this study are available from the corresponding author.

## Discussion

The key finding of this study is that the majority (100% of the included patients in Stage II and 80% of the included patients in Stage III) of older patients can independently fill out the digital visual P-BAS at home without any assistance of a health care professional. In order to improve the accessibility of the original P-BAS, designed for already hospitalized patients, the picture-based tool has been digitalized. When a patient indicated that the goal was applicable, the additional questions followed. Given that this tool was originally developed for hospitalized patients, where researchers explained the tool to the patients and that the tool has been digitalized for the outpatients, clarity was the main issue found. The pictures accompanying the goals (Appendix 1) may contribute to making the P-BAS more suitable for patients with low health literacy.

A limitation of the study is that a relatively small sample size of patients was used. Despite the sample size, the cognitive interview process was able to identify different types of issues with the draft questionnaires resulting in amendments and improvements. This may have improved the quality of the questionnaire, as a decrease in the number of problems was reported in Stage II. In addition, the cognitive interviews were conducted, with a similar group of patients who will eventually use the P-BAS. This increases the applicability of the tool.

Another limitation of this study is that there were no cognitive interviews in Stage III of the study. No clear insights are available about the reduction of problems between Stage II and III. Despite this was not done, the researchers did call patients in Stages II and III to evaluate the process of completing the questionnaire. Cognitive interviews were conducted to assess the extent to which older patients were able to independently fill in the P-BAS. Although there are several opponents that advise not to use a digital questionnaire with older outpatients, there are several advantages and positive outcomes that should be mentioned: firstly, the digital P-BAS assists older patients in identifying their goals and can thereby facilitate the process of shared decision making. Secondly, it turns out in practice that patients are capable of filling out a digital questionnaire, whether or not with the help of relatives. This emerges from the fact that patients were able to open the digital questionnaire, and 80% of the patients in stage III successfully completed the digital visual P-BAS, but that also the identified problems reduced after the improvements were implemented.

Thirdly, the patients’ feedback on the evaluating questions in stage III were that the digital visual P-BAS was “easy to fill in independently” and “the instructions and the goal of the questionnaire are clear.” The cognitive interviews actually showed that by filling in the digital questionnaire a meaningful conversation and/or discussion was started. For example: “No, staying alive is no longer my main goal. I think that the quality of my life is now more important.”

The P-BAS focuses on goals of older patients visiting the outpatient clinic. Many existing instruments and tools used in shared decision making focus on (1) the conversation between healthcare professional and patient, (2) informing the patient about treatment options, or (3) assessing the current state of mind of the patient regarding their physical, social and mental functioning. However, there are a limited number of tools that actually identify the patient’s goals regarding their future hospital care. This is despite the fact that in the literature (Lenzen et al., 2018; N. P. Vermunt et al., 2018) the steps of “defining goals” are considered to be the first step in the shared decision-making process. Using the P-BAS in a digital visual form may be more efficient and less time consuming, as it allows patients to complete the P-BAS independently and remotely. This could result in the patient being better prepared before meeting the healthcare professional. Besides, healthcare professionals can analyze the P-BAS outcomes and discuss the outcomes before or during the consult with their patient. This contributes to the most personalized care for the patient.

## Conclusion and Implications

Cognitive interviews provided insight into patients’ interpretation and understanding of the digital visual P-BAS, resulting in a feasible questionnaire whereby patients are able to independently fill in the digital visual P-BAS at home without any assistance. The digital visual P-BAS might be of added value for patients and contributes to the process of shared decision making, by making it possible to take the goals of the patient into account when choosing a treatment. Whereby in some cases refraining treatment also would become an acceptable option. Based on the individual goals and preferences of older patients the added value of a specific treatment choice can be assessed as well. Future research includes the development of the P-BAS questionnaire by evaluating the reliability and factor structure.

## Supplemental Material

sj-docx-1-ggm-10.1177_23337214241230159 – Supplemental material for Feasibility study of the Digital Patient Benefit Assessment Scale (P-BAS): A Digital Tool to Assess Individual Patient Goals Click here for additional data file. Supplemental material, sj-docx-1-ggm-10.1177_23337214241230159 for Feasibility study of the Digital Patient Benefit Assessment Scale (P-BAS): A Digital Tool to Assess Individual Patient Goals by Rozemarijn A.L. van Erp, Sophia E. de Rooij, A.N. Machteld Wymenga, A.V.C.M. Elgun Zeegers and Job van der Palen in Gerontology and Geriatric Medicine

sj-docx-2-ggm-10.1177_23337214241230159 – Supplemental material for Feasibility study of the Digital Patient Benefit Assessment Scale (P-BAS): A Digital Tool to Assess Individual Patient Goals Click here for additional data file. Supplemental material, sj-docx-2-ggm-10.1177_23337214241230159 for Feasibility study of the Digital Patient Benefit Assessment Scale (P-BAS): A Digital Tool to Assess Individual Patient Goals by Rozemarijn A.L. van Erp, Sophia E. de Rooij, A.N. Machteld Wymenga, A.V.C.M. Elgun Zeegers and Job van der Palen in Gerontology and Geriatric Medicine
