# Supplementary material for: Feasibility study of the Digital Patient Benefit Assessment Scale (P-BAS): A Digital Tool to Assess Individual Patient Goals
Source: Gerontol Geriatr Med. 2024 Feb 7;10:23337214241230159. doi: 10.1177/23337214241230159 (PMC10848793; doi:10.1177/23337214241230159)
Supplement: sj-docx-1-ggm-10.1177_23337214241230159 – Supplemental material for Feasibility study of the Digital Patient Benefit Assessment Scale (P-BAS): A Digital Tool to Assess Individual Patient Goals [file sj-docx-1-ggm-10.1177_23337214241230159.docx]

**Appendix 1:** **Overview of included goals and accompanying pictures in the digital visual P-BAS**

| **Goal number Description in Dutch English translation** | | |
| --- | --- | --- |
| Doel 1 | Mij beter voelen | Feeling better |
| Doel 2 | Energie | Energy |
| Doel 3 | Pijn | Pain |
| Doel 4 | Ontlasting | Defecation |
| Doel 5 | Kortademigheid | Short of breath |
| Doel 6 | Lopen | Walking |
| Doel 7 | Eetlust | Appetite |
| Doel 8 | Duidelijkheid over wat ik mankeer | Clarity about disease |
| Doel 9 | Genezen/ziekteproces afremmen | Cure/slow down disease process |
| Doel 10 | In leven blijven | Staying alive |
| Doel 11 | Van het leven genieten | Enjoying life |
| Doel 12 | Boodschappen | Doing groceries |
| Doel 13 | Wassen en aankleden | Washing and getting dressed |
| Doel 14 | Tuinieren | Gardening |
| Doel 15 | Sporten | Sporting |
| Doel 16 | Hobby’s | Hobbies |
| Doel 17 | Autorijden | Driving a car |
| Doel 18 | Uitstapjes maken | Going on trips |
| Doel 19 | Visite | Paying visits |
| Doel 20 | Thuis blijven wonen | Keep living at home |
| Doel 21  Doel 22 | Zelfstandigheid | Independency |
|  | Zelf doel bepalen Set own goal | |


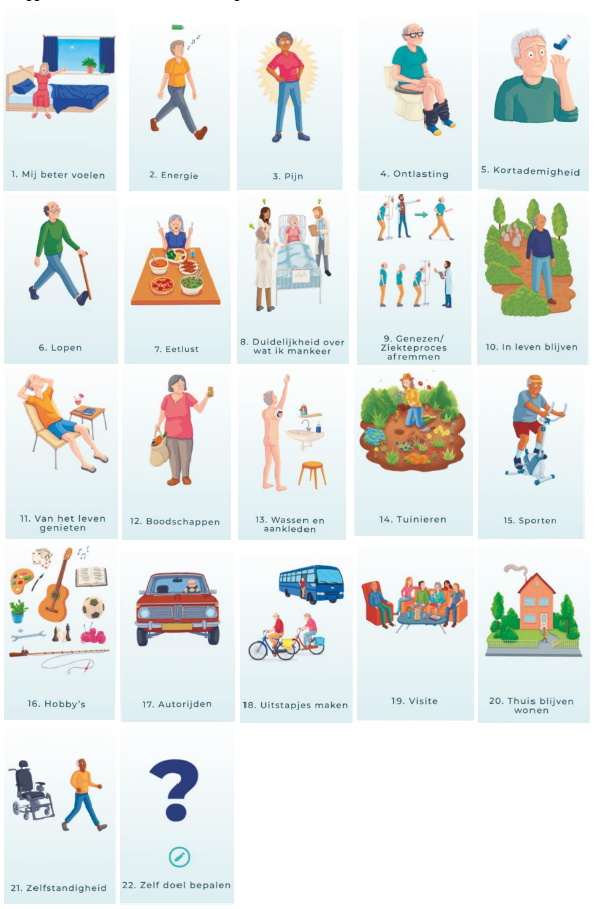


**Appendix 2: Classification table problems stage 1**

| **Frequency of categories** | | | | | | | | | |
| --- | --- | --- | --- | --- | --- | --- | --- | --- | --- |
| **Goal** | **Clarity** | **Knowledge** | **Assumptions** | **Response categories** | **Sensitivity** | **Instructions** | **Formatting** | **Total** |  |
| 1. Feeling better | 14 | - | 3 | 2 | - | 3 | 1 | 23 |  |
| 2. Energy | 2 | - | 1 | 1 | - | 1 | - | 5 |  |
| 3. Pain | 8 | - | 5 | 5 | - | - | - | 18 |  |
| 4. Defecation | 10 | - | 5 | 2 | 1 | - | - | 18 |  |
| 5. Shortness of breath | 2 | - | - | - | - | 1 | 1 | 4 |  |
| 6. Walking | - | - | - | - | - | 1 | - | 1 |  |
| 7. Appetite | 4 | - | - | - | - | - | 1 | 5 |  |
| 8. Clarity about disease | 8 | 2 | 4 | 4 | - | - | - | 18 |  |
| 9. Cure/slow down disease process | 6 | 1 | 4 | 3 | 3 | 1 | - | 18 |  |
| 10. Staying alive | 1 | - | 1 | 2 | 6 | 1 | - | 11 |  |
| 11. Enjoying life | 2 | - | - | - | - | 1 | - | 3 |  |
| 12. Doing groceries | 1 | - | 1 | - | - | - | - | 2 |  |
| 13. Washing and getting dressed | 2 | 1 | 1 | - | - | - | - | 4 |  |
| 14. Gardening | 1 | 2 | 2 | - | 1 | - | - | 6 |  |
| 15. Sporting | 2 | - | 1 | 6 | - | - | - | 9 |  |
| 16. Hobbies | 1 | 1 | - | - | 1 | 1 | - | 4 |  |
| 17. Driving a car | - | - | - | - | - | - | - | 0 |  |
| 18. Going on trips | 2 | - | - | 5 | - | - | - | 7 |  |
| 19. Paying visits | 1 | - | - | 8 | - | 2 | - | 11 |  |
| 20. Keep living at home | - | - | - | 1 | 1 | - | - | 2 |  |
| 21. Independency | - | - | - | - | - | - | - | 0 |  |
| 22. Set own goal | 5 | 1 | 4 | - | 1 | - | 1 | 12 |  |
| **Total** | **72** | **8** | **32** | **39** | **14** | **12** | **4** | **181** |  |

**Appendix 3: Classification table problems stage 2**

| **Frequency of categories** | | | | | | | | | |
| --- | --- | --- | --- | --- | --- | --- | --- | --- | --- |
| **Goal** | **Clarity** | **Knowledge** | **Assumptions** | **Response categories** | **Sensitivity** | **Instructions** | **Formatting** | **Total** |  |
| 1. Feeling better | 2 | 1 | 1 | 1 | 1 | 1 | 1 | 5 |  |
| 2. Energy | 3 | 1 | 1 | 1 | - | - | - | 6 |  |
| 3. Pain | 4 | 1 | 1 | 1 | - | - | - | 7 |  |
| 4. Defecation | 2 | - | 2 | - | - | - | - | 4 |  |
| 5. Shortness of breath | 4 | - | - | 2 | - | - | - | 6 |  |
| 6. Walking | - | - | 1 | 2 | - | - | - | 3 |  |
| 7. Appetite | 1 | - | - | 1 | - | - | - | 4 |  |
| 8. Clarity about disease | 3 | - | - | 1 | - | - | - | 4 |  |
| 9. Cure/slow down disease process | 5 | - | - | 1 | - | - | 1 | 7 |  |
| 10. Staying alive | - | - | - | 2 | - | - | - | 2 |  |
| 11. Enjoying life | 2 | - | 1 | - | - | - | - | 3 |  |
| 12. Doing groceries | 4 | - | - | - | - | - | - | 4 |  |
| 13. Washing and getting dressed | 1 | - | - | - | - | - | - | 1 |  |
| 14. Gardening | 1 | - | - | - | - | - | - | 1 |  |
| 15. Sporting | 1 | - | - | - | - | - | - | 1 |  |
| 16. Hobbies | - | - | 2 | - | - | - | - | 2 |  |
| 17. Driving a car | - | 1 | 1 | - | - | - | - | 2 |  |
| 18. Going on trips | 1 | - | 1 | 1 | - | - | - | 3 |  |
| 19. Paying visits | 1 | - | - | 3 | - | - | - | 4 |  |
| 20. Keep living at home | - | - | 2 | - | - | - | - | 2 |  |
| 21. Independency | 1 | - | - | - | - | - | - | 1 |  |
| 22. Set own goal | - | - | - | - | - | 1 | - | 1 |  |
| **Total** | **36** | **3** | **12** | **14** | **1** | **2** | **2** | **70** |  |
